# Nearly Complete Genome Sequences of Eight Rabies Virus Strains Obtained from Domestic Carnivores in the Democratic Republic of the Congo

**DOI:** 10.1128/MRA.01109-21

**Published:** 2022-01-06

**Authors:** Patient Pati Pyana, Céline Mbilo, Julien Lannoy, Simon Bonas, Bobo Luntadila, Jean Baptiste Kabongo, Ipos Ngayi Lukusa, Leonard Ntunuanga, Jakob Zinsstag, Stephanie Mauti, Laurent Dacheux

**Affiliations:** a Institut National de Recherche Biomédicale (INRB), Laboratoire de la Rage, Gombe, Kinshasa, Democratic Republic of the Congo; b Université Pédagogique Nationale de Kinshasa, Kinshasa, Democratic Republic of the Congo; c Swiss Tropical and Public Health Institute, Basel, Switzerland; d University of Basel, Basel, Switzerland; e Institut Pasteur, Université de Paris, Unit Lyssavirus Epidemiology and Neuropathology, National Reference Center for Rabies, WHO Collaborating Center for Reference and Research on Rabies, Paris, France; f Division Provinciale de Pêche et Elevage, Matadi, Province du Kongo Central, Democratic Republic of the Congo; DOE Joint Genome Institute

## Abstract

In this report, we describe eight nearly complete genome sequences of rabies virus strains collected in the Democratic Republic of the Congo from domestic carnivores in 2017 and 2018. All of them clustered into a specific phylogroup among the Africa 1b lineage in the Cosmopolitan clade.

## ANNOUNCEMENT

Rabies is the prototype of a neglected and tropical zoonotic disease, affecting poor and rural areas in Asia and Africa. To date, nearly 59,000 human cases of rabies are estimated worldwide each year, mainly due to transmission from dogs (1). Rabies virus (RABV) is the principal etiological agent of rabies, an acute and almost always fatal form of encephalomyelitis which can affect potentially all mammalian species. This virus belongs to the prototype species Rabies lyssavirus within the genus *Lyssavirus*, family *Rhabdoviridae* (order *Mononegavirales*) (2). Dog rabies is endemic in the Democratic Republic of the Congo (DRC), similarly to other sub-Saharan countries (3, 4). However, data available about the genetic diversity of RABV strains circulating in this country still remain extremely limited (5).

**FIG 1:**
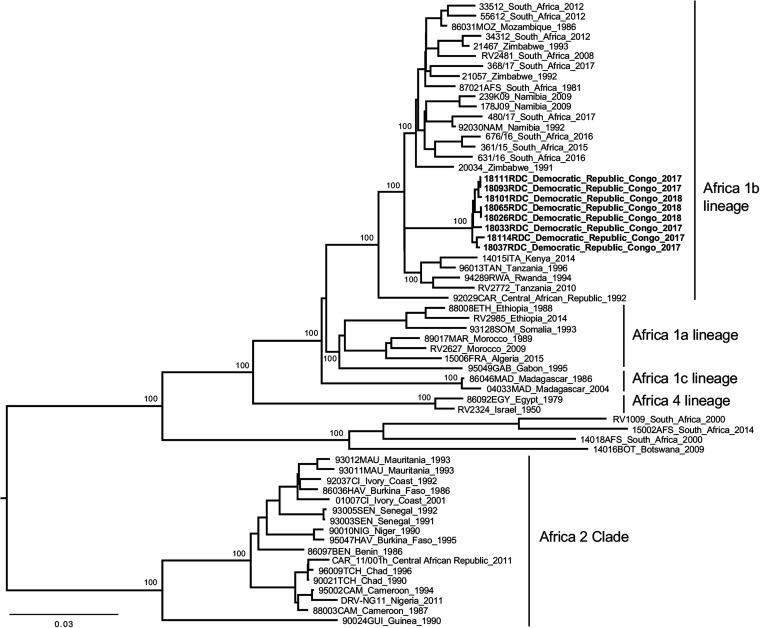
Phylogenetic analysis of the eight RABV strains from the Democratic Republic of the Congo and different representative African strains. The tree was based on the nearly complete genome sequences (11,786 to 11,807 nt) and constructed using the maximum‐likelihood approach based on the generalized time‐reversible model proportion of invariable sites plus the gamma‐distributed rate heterogeneity (GTR + I + Γ4), utilizing subtree pruning and regrafting (SPR) of branches, as estimated using PhyML 3.0 (12) with Smart Model Selection (http://www.atgc-montpellier.fr/phyml-sms/). The robustness of individual nodes was estimated using 100 bootstrap replicates. Only bootstrap values of ≥90 are indicated. The scale bar indicates nucleotide substitutions per site.

In this study, brain samples were collected from seven dogs and one cat suspected of being infected with rabies that originated from different health sanitary zones of Kongo Central Province in the DRC in 2017 and 2018 (Table 1). All these samples were confirmed positive by direct fluorescence antibody test (FAT) (6) at the Institut National de Recherche Biomédicale (INRB) in Kinshasa. Total RNA was extracted from one brain biopsy sample (approximatively 0.5 cm^3^) from each animal using the Direct-zol RNA miniprep kit (Zymo Research), following the manufacturer’s instructions and performed in the rabies laboratory of INRB in Kinshasa. The RNA was then purified using Agencourt RNAClean XP beads (Beckman Coulter) at a ratio of 1:1.8, following the manufacturer’s instructions, without the last resuspension step in nuclease-free water for half of these samples (Table 1). For the other half, RNA was eluted in 30 to 50 μL of nuclease-free water, and 20 μL was deposited in a 96-well plate (RNAstable; Biomatrica), before overnight air-drying in a laminar flow hood, following the manufacturer’s instructions (Table 1). Dried RNAs in an RNAstable 96-well plate or on beads were shipped to Institut Pasteur, Paris, France, at ambient and cold temperature with ice packs, respectively, and resuspended in 30 μL nuclease-free water. The eight RNA samples were processed for next-generation sequencing (NGS) as previously described (7–9). Briefly, an rRNA depletion step was first carried out using Terminator 5′‐phosphate‐dependent exonuclease (Epicentre Biotechnologies), following the instructions of the manufacturer. After purification, the depleted RNA was reverse transcribed into cDNA using random primers and Superscript III reverse transcriptase (Invitrogen), according to the manufacturer’s instructions, and double-stranded DNA (dsDNA) was synthesized as already described (7–9). Finally, dsDNA libraries were constructed using the Nextera XT kit (Illumina) and sequenced using a 2 × 150-nucleotide (nt) paired-end strategy on the NextSeq 500 platform (7–9). NGS data were analyzed using *de novo* assembly and mapping (both using CLC Assembly Cell, Qiagen), with a dedicated workflow built on the Institut Pasteur Galaxy platform (7–10). Contig sequences were assembled to produce the final consensus genome using Sequencher 5.2.4 (Gene Codes Corporation). The quality and accuracy of the final genome sequences were checked after a final mapping step of the original cleaned reads and visualized using Tablet (11). Maximum likelihood (ML) phylogenetic analysis was performed on the nearly complete genome sequences (11,786 to 11,807 nt) of the eight RABV strains and different representative African strains using PhyML (12), after a multiple alignment step performed using ClustalW 2.1 (13), implemented in the Institut Pasteur Galaxy platform (10). The ML phylogenetic tree was visualized using FigTree (http://tree.bio.ed.ac.uk/) (Fig. 1). All tools were run with default parameters unless otherwise specified.

**TABLE 1:** Description of the genome sequences of the eight rabies viruses obtained from domestic carnivores in Kongo Central Province of the Democratic Republic of the Congo

Virus name	Code	Host	Animal status	Location	Collection date (day-mo-yr)	Support^*a*^	Total no. of reads	No. of mapped reads (%)	Avg coverage (×)	Genome length (nt)	GC content (%)	GenBank accession no.	SRA accession no.
18033RDC	1Cn3	Dog	Owned	Matadi	28-Oct-17	Plate	2,221,528	92,167 (4.1)	1,141.6	11,923	46	OK317992	SRX12433974
18065RDC	7Cn41	Dog	Owned	Boma	2-Jul-18	Beads	4,028,176	8,353 (0.2)	103.5	11,868^*b*^	46	OK317993	SRX12433975
18026RDC	7Cn68	Dog	Owned	Boma	16-Sep-18	Beads	3,724,300	92,361 (2.5)	1,148.4	11,899^*b*^	46	OK317994	SRX12433976
18093RDC	9Cn11	Dog	Unowned	Muanda	25-Nov-17	Beads	4,734,998	2,414,598 (51)	29,967.5	11,923	46	OK317995	SRX12433977
18101RDC	9Cn22	Dog	Unowned	Muanda	27-Jun-18	Plate	1,515,480	10,631 (0.7)	132.2	11,882^*b*^	46	OK317996	SRX12433978
18111RDC	9Ct9	Cat	Owned	Muanda	4-Nov-17	Plate	3,635,804	20,729 (0.6)	257.8	11,877^*b*^	46	OK317997	SRX12433979
18037RDC	11Cn1	Dog	Unowned	Mbanza-Ngungu	29-Nov-17	Plate	2,332,676	45,426 (1.9)	566	11,886^*b*^	46	OK317998	SRX12433980
18114RDC	KN	Dog	Owned	Kinshasa (Ngaliema)	2-Feb-18	Beads	2,561,938	85,775 (3.3)	1,066.9	11,923	46	OK317999	SRX12433981

a Dry RNA was stored and shipped in plates (RNAstable 96-well plate) or on beads (Agencourt RNAClean XP).

b Genome sequence with incomplete 5′ untranslated region (UTR).

The genome sequences presented the five canonical genes encoding the nucleoprotein (N; 1,353 nt, 450 amino acids [aa]), phosphoprotein (P; 894 nt, 297 aa), matrix protein (M; 609 nt, 202 aa), glycoprotein (G; 1,575 nt, 524 aa), and RNA polymerase (L; 6,384 nt, 2,127 aa) (Table 1). The leader and trailer sequences, when complete, were 58 and 70 nucleotides long, respectively (checking done after alignment with genetically close and available complete genomes [Fig. 1]) (Table 1). The transcription initiation (TI) signal AACA and the transcription termination polyadenylation (TTP) sequences TGA_7_ were observed for all the genes, except for the G gene, which presented the AGA_7_ motif for TTP. The nucleotide identity between the eight genome sequences, determined using Ident and Sim software implemented in the Sequence Manipulation Suite (https://www.bioinformatics.org/sms2/ident_sim.html) (14), was high (>98.9%), and genetic analysis confirmed that they clustered together in lineage Africa 1b within the Cosmopolitan clade (Fig. 1) (15).

### Data availability.

The nearly complete genome sequences of the eight rabies viruses from the Democratic Republic of the Congo were deposited at GenBank under the accession numbers OK317992 to OK317999 and the BioProject accession number PRJNA767799.
